# Fragmentation of nucleoli in senescent cancer cells is associated with increased levels of polyadenylated transcripts derived from noncoding regions of rDNA units

**DOI:** 10.1007/s00249-025-01773-9

**Published:** 2025-06-23

**Authors:** Jana Sochorová, Emilie Lukášová, Eva Volfová Polanská, Kateřina Řehůřková, Aleš Kovařík

**Affiliations:** Institute of Biophysics, Czech Academy of Sciences, Královopolská 135, Brno, 61200 Czech Republic

**Keywords:** Irradiation, Senescence, Cancer cell, rDNA, Nucleolus

## Abstract

In this study, we investigated the behavior of rDNA loci in senescent MCF-7 mammary cancer cells induced by gamma irradiation. To analyze changes in nucleolar structure we used rDNA-FISH and immunohistochemical staining with fibrillarin and UBF transcription factor. The expression levels of rDNAs and nucleolar proteins were determined by RNA-seq of total and poly-A libraries. The cytological and molecular parameters of nucleoli were monitored throughout the 7-day interval following irradiation. Senescent cells exhibited a higher proportion of smaller nucleoli as compared to cycling cells, indicating nucleolar fragmentation. The rDNA copy number and expression of rDNA variants remained stable in cycling and senescent cells. However, the levels of polyadenylated rRNA species derived from external (5′ETS) and internal (ITS1) rDNA spacers tend to increase (c.2 fold) following irradiation. At the protein level, senescent cells showed decreased levels of fibrillarin and UBF transcription factor while localization of both proteins in the nucleolus was not impaired. We conclude that withdrawal from cell cycle does not change expression patterns of rDNA variants. However, defects in rRNA processing may lead to fragmentation of nucleoli in senescent cells.

**Supplementary Information:**

The online version contains supplementary material available at 10.1007/s00249-025-01773-9.

## Introduction

**Fig. 1:**
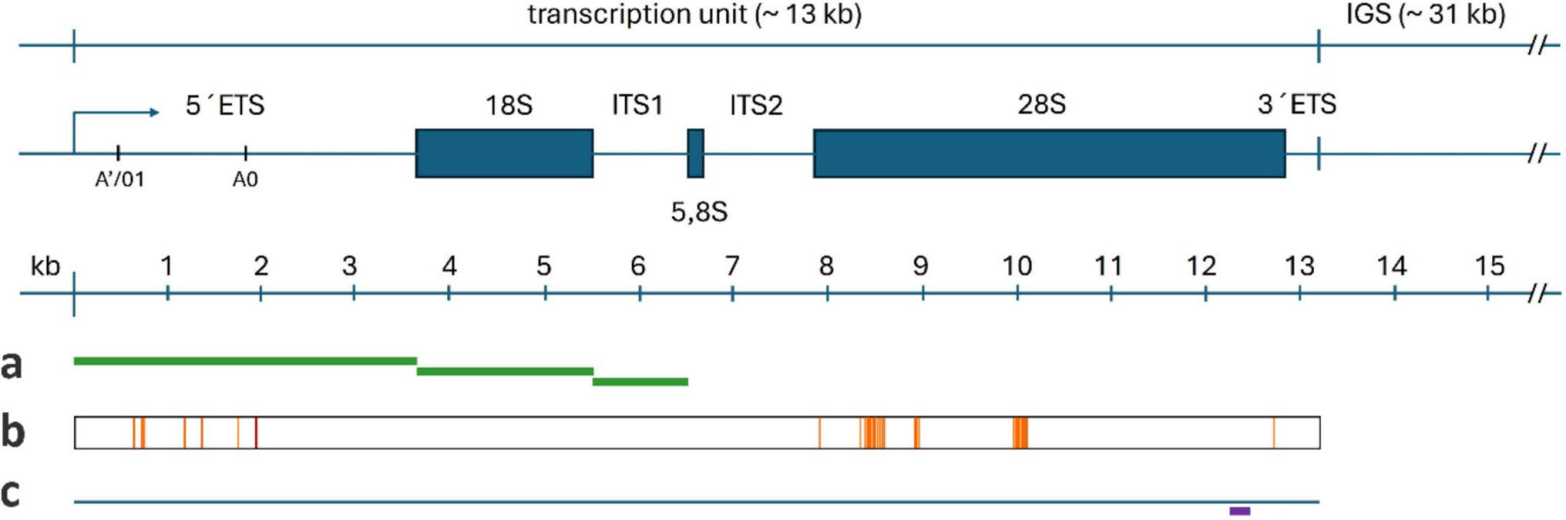
The 47S rDNA unit structure and the position of sequences used for analyses. **a** Regions used for the transcriptomic analysis of rDNA. **b** Position of polymorphic sites used for analysis of rDNA variants. **c** Site of a FISH probe hybridization

In humans, major rDNA is encoded by 5 loci at acrocentric chromosomes (chr 13, 14, 15, 21 and 22) totally comprising about 300 units. Primary pre-rRNA transcripts (47S rRNA) generated by RNA polymerase I (Pol I) contain coding regions (encoding 18S, 5.8S and 28S rRNA molecules) and the noncoding intergenic (IGS, ETS) and intragenic (ITS) spacers (Fig. 1). 47S rRNA undergoes complicated processing before the functional rRNA molecules are incorporated into the ribosomes (An et al. 2024). The synthesis of 47S rRNA is also regulated by the mTOR signaling pathway due to the modulating basal transcription factors including UBF, which maintains the euchromatic state of rDNA chromatin (Sanij and Hannan 2009). It is estimated that no more than 50% of the ~ 300 copies of the rRNA genes are active at any given time. How and why a majority of rDNA are silenced is unclear, but epigenetic mechanisms, such as DNA methylation, seem to be involved (Grummt and Pikaard 2003). Recent studies indicate that in addition to controlling rRNA synthesis, rDNA silencing plays an essential role in maintaining the genetic stability of rDNA repeats and influences cellular events such as aging and senescence (Ganley and Kobayashi 2014; Symonova 2019). Both Pol I transcription and 47S rRNA processing occurs in the nucleolus which is a membrane-less nuclear organelle composed of phase-separated substructures known as the fibrillar center (FC), dense fibrillar component (DFC), and granular component (GC). At the interface between the FC and the DFC, rDNA transcription generates 47S rRNA, which subsequently undergoes chemical modification and cleavage in DFC. Pre-rRNA processing is monitored by the nuclear RNA surveillance system where aberrant processing intermediates are appended with a short poly(A) tail that activates the nuclear exosome for subsequent degradation (Slomovic et al. 2006). Fibrillarin is a major component of DFC of nucleoli involved in pre-rRNA processing of the nascent 47S pre-rRNA (Yao et al. 2019) (Zhang et al. 2024). It interacts with rRNA and harbors catalytic 2-*O*-ribosyl methylation RNA activity important for protein translation at ribosomes. FBL overexpression contributes to tumorigenesis and is associated with poor survival in patients with breast cancer (Marcel et al. 2013).

Different kinds of stresses, such as nutrient starvation, hypoxia, heat shock, chemical suppression, and genetic impairments of ribosome biogenesis factors can disturb the elaborately regulated biogenesis of ribosomes in animals (Blanchard et al. 2019; Mayer and Grummt 2005; Tiku and Antebi 2018). Perturbations in nucleolar functions have been associated with several diseases, such as cancer and progeria (Buchwalter and Hetzer 2017; Dahl et al. 2007; Kasselimi et al. 2022; Misteli and Scaffidi 2005). The gamma irradiation caused nucleolar re-positioning over time and changed several morphological parameters, including the size of the nucleolus and the area of individual UBF1-positive foci (Franek et al. 2016). Irradiation may also influence epigenetic landscapes of Pol I promoters (Stixová et al. 2019). Genomes of replicatively senescent cells undergo global epigenetic changes leading to gene silencing and chromatin changes (Cecco et al. 2013). Accumulation of rRNA precursors in senescent cells was reported following oncogenic transformation and toxic chemical treatments (Szaflarski et al. 2022).

The motivation of this study was to determine if nucleolar structure and rRNA transcriptomes are influenced by a switch from cycling to senescent cells. To address these questions, we used the epithelial mammary carcinoma MCF-7 cells known to efficiently undergo senescence following gamma irradiation (Bravata et al. 2015; Lukasova et al. 2018). We determined the localization of nucleolar proteins by immunohistochemical staining combined with confocal microscopy. We generated RNA-seq libraries from different intervals following gamma irradiation and analyzed the rRNA transcriptomes. The list of experiments carried out in this study is given in supplementary Tab. S1. Evidence was obtained for changes in the abundance of poly-adenylated rRNA transcripts derived from noncoding parts of rDNA units.

## Results

### Distribution of rDNA and nucleolar proteins in control and senescent MCF-7 cells

**Fig. 2:**
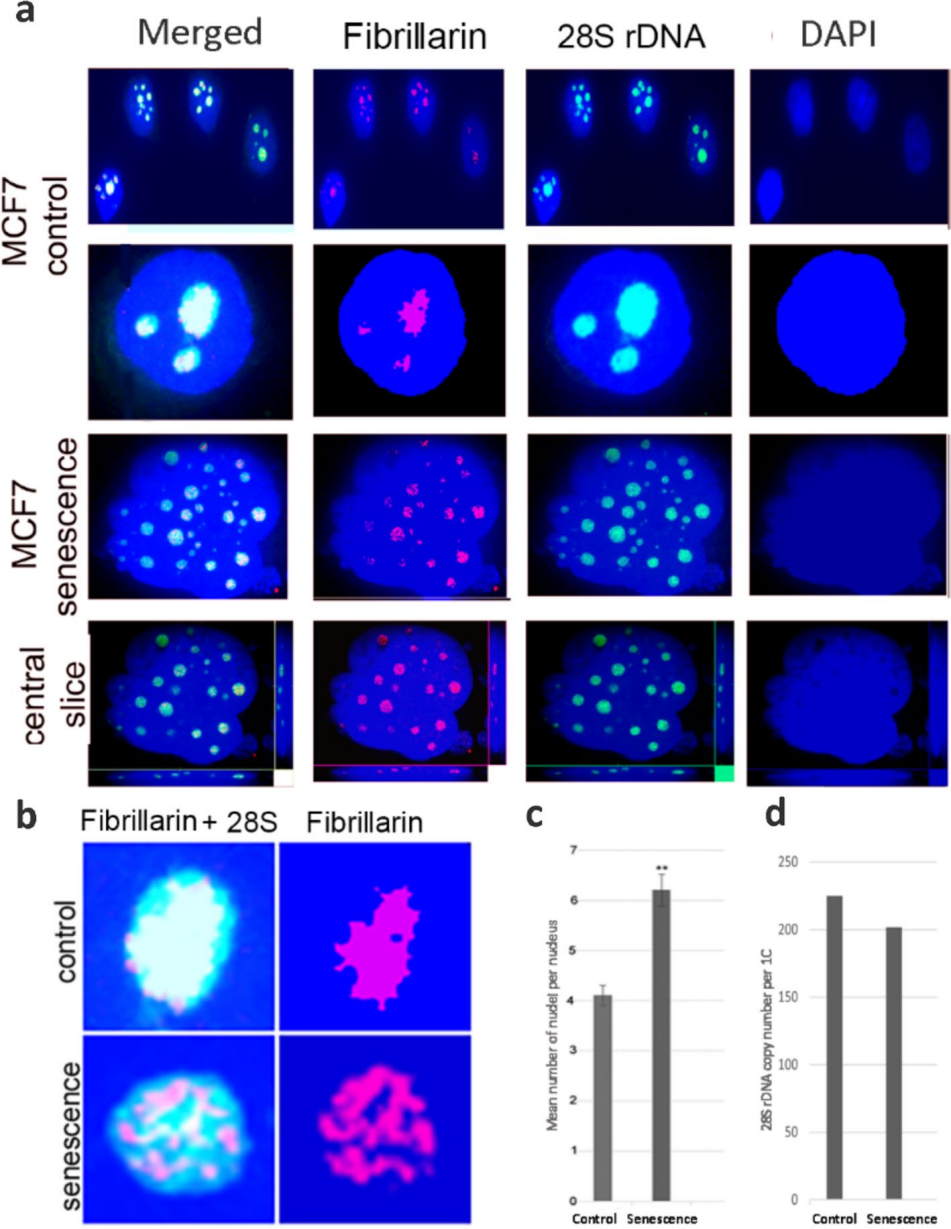
Cytogenetic and molecular analysis of rDNA loci in MCF-7 cells following gamma irradiation. **a** Immunostaining of control and irradiated interphase cells with an antibody to fibrillarin (in red) followed by in situ hybridization with the 28S rDNA probe (green). **b** Colocalization of fibrillarin and 28S rRNA genes. **c** Nucleoli counts in control cycling and senescent (168 h post irradiation interval) cells. **d** 28S rDNA copy number in control and senescent cells

We investigated the changes in nucleoli number and morphology and the nucleolar protein distribution in gamma-irradiated MCF-7 cells. The senescent phenotype was manifested in most cells at the 168 h interval following irradiation by positive staining with beta galactosidase and reduced LBR marker expression (Lukasova et al. 2017). To detect the nucleoli we used combined fluorescent in situ hybridization of 28S rDNA (rDNA–FISH) and fibrillarin immunostaining of cells with an antibody to fibrillarin (Fig. 2a).

Overlap of rDNA–FISH and immunostaining was apparent in both cycling and senescent cells. Nucleoli in senescent cells often display prolonged ragged shape. In contrast, cycling cells displayed regular round shape of nucleoli. In cycling MCF-7 cells, fibrillarin signal occupied central part of the nucleolus, and in large nucleoli, it filled the whole nucleolus. In contrast, senescent nucleoli often showed clustered fibrillarin signals across the nucleoli with negatively stained regions in between (Fig. 2b). We counted the number of 28S rDNA/fibrillarin signals (representing nucleoli) in control and senescent cells. The values were obtained from four biological replicates and counting more than 100 nuclei in each group. A mean number of nucleoli per nucleus in control nonirradiated and senescent MCF-7 cells was 4.2 and 6.2, respectively (*t* test, 2-side, *N* = 400, *p* < 0.01) (Fig. 2c). The rDNA copy number of rDNA variants remained stable in cycling and senescent cells (Fig. 2d).

**Fig. 3:**
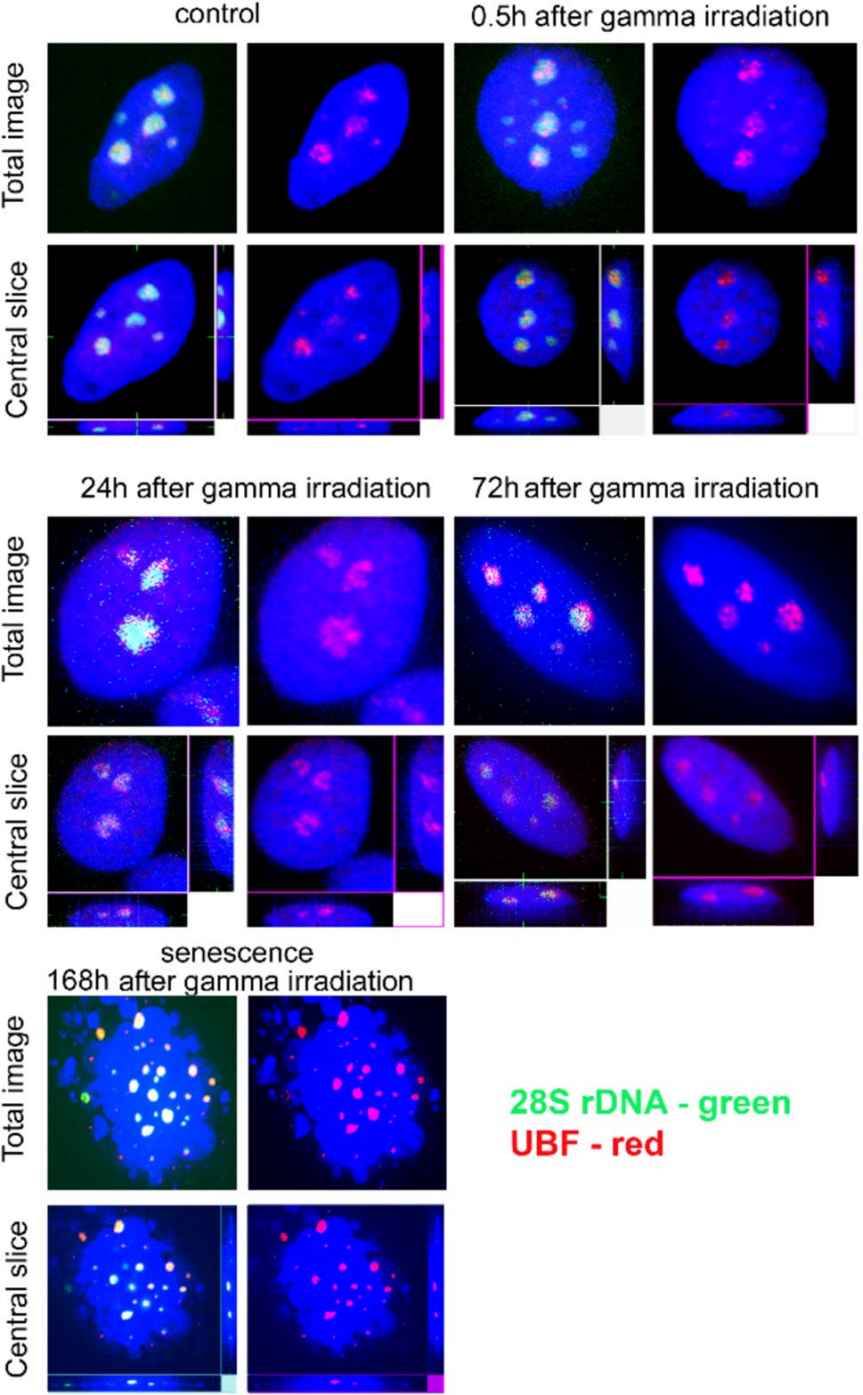
Immunostaining of control and irradiated MCF-7 cells with an antibody to the UBF transcription factor. UBF and 28S rDNA signals are visualized in red and green fluorescence, respectively. Staining of cells was carried out in cycling cells and at different intervals following irradiation

UBF is a critical upstream binding factor, which functions in the regulation of euchromatic rDNA chromatin and transcription activation. We analyzed the distribution of UBF in cycling and senescent cells. Combining 28S rDNA–FISH and immunostaining with an antibody to UBF revealed colocalization of 28S rDNA and UBF in both cells (Fig. 3). The UBF signals were visible in large nucleoli while smaller nucleoli often showed little or no signals. Colocalization of 28S rDNA and UBF was visible even in severely damaged cells displaying blebbing.

### Expression of nucleolar and lamina proteins in cycling and senescent cells

**Fig. 4:**
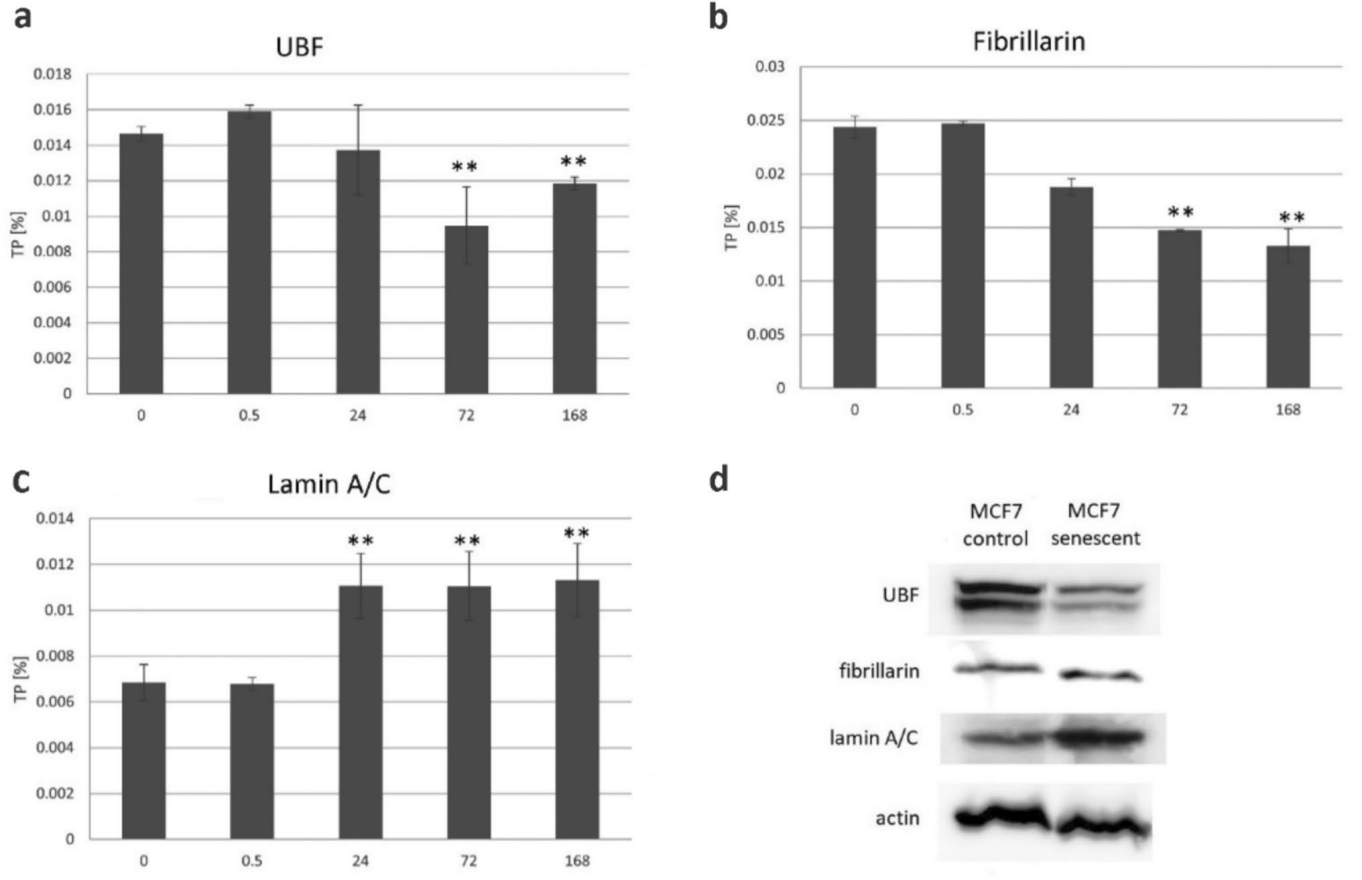
Expression analysis of nucleolar proteins in control and senescent MCF-7 cells. **a**, **b** mRNA levels of fibrillarin and UBF in cycling cells and 0.5, 24, 72 and 168 h after the gamma irradiation. **c** Expression of lamin A/C was used as a marker of senescence. The data were collected from three biological experiments and the values were averaged (Tab. S2). **d** Western blot analysis of fibrillarin, UBF and lamin A/C in protein extracts from control cycling and senescent (168 h interval) cells. Contrasting trends of UBF and lamina A/C after the irradiation

Expression of nucleolar (UBF and fibrillarin) and the nuclear lamina (Lamin A/C) proteins were analyzed at the protein (Western blot) and mRNA (transcriptomes) levels. The mRNA levels of respective proteins were determined by counting of reads in poly-A transcriptomes at different time point intervals following irradiation (0.5, 24, 72 and 168 h) and expressed as a percentage (Fig. 4a–c and Supplementary Table S2). The UBF and fibrillarin mRNA levels showed a descending trend following irradiation. UBF mRNA started to decrease from 24 h following irradiation. A similar trend showed fibrillarin reaching only 40% of the mRNA levels in the 168 h interval following irradiation compared to cycling cells. In contrast to nucleolar proteins, lamin A/C showed increased levels in senescent cells. On Western blot, UBF showed reduced signals in senescent cells while those of lamin A/C increased (Fig. 4d) consistent with studies of (Mayer and Grummt 2006; Solovei et al. 2013). The fibrillarin protein levels remained stable.

### Analysis of single nucleotide rDNA variants and copy number

**Fig. 5:**
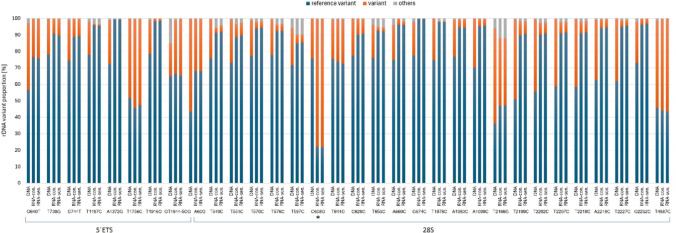
Genomic and transcriptomic analysis of rDNA variation. Single nucleotide variants were called in the 5’ETS and 28S rDNA subregions in mapped reads (see Supplementary Table S3). Representation of variants in polymorphic sites was similar between control and senescent cells. Asterisk indicates silencing of a genetically dominant variant

It is known that human rDNA is heterogeneous both at the sequence and copy number levels (Hall et al. 2021). We wondered whether the heterogeneity is affected by the transition from cycling to senescent cells. We first analyzed single nucleotide variation (SNV) in genomic DNA and the transcriptomes. Illumina reads were mapped to the 5′ETS and 28S reference sequences, and variants were called at the 20% (genomic) and 1% (transcriptomic cutoffs, respectively. At the genomic level, there were eight and twenty-four prominent SNPs in the 5′ETS and 28S rDNA, respectively (Fig. 1b, Fig. 5 and supplementary Tab. S3). The difference in the number of polymorphic sites between both subregions was not significant (Chi square 0.3257, *p* = 0.568). In the transcriptomes, abundant genomic variants were expressed while the minor variants were frequently but not absolutely suppressed. An exception was a minor ‘G’ variant at the position 608 of the 28S rRNA gene (C608G) which appeared to be more strongly expressed than the genetically dominant ‘C’ variant. There were no differences between cycling and senescent cells (Fig. 5) and between transcriptomes derived from different libraries (not shown). We conclude that the 28S RNA and 5’ETS transcripts are heterogeneous in MCF-7 line, while the heterogeneity is not influenced by transition from cycling cells to senescence.

Increased number of 28S rDNA signals in senescent cells can potentially be explained by amplification of rDNA following double-strand breaks induction (Ganley et al. 2009). We therefore calculated rDNA copy number in control cycling and senescent cells from the genome proportions (Fig. 2d and Supplementary Table S4). It is evident that that the rDNA copy numbers were similar between both groups and matched to that of the MCF-7 line average.

### Senescent cells exhibit increased levels of polyadenylated rRNA transcripts derived from noncoding rDNA spacers

**Fig. 6:**
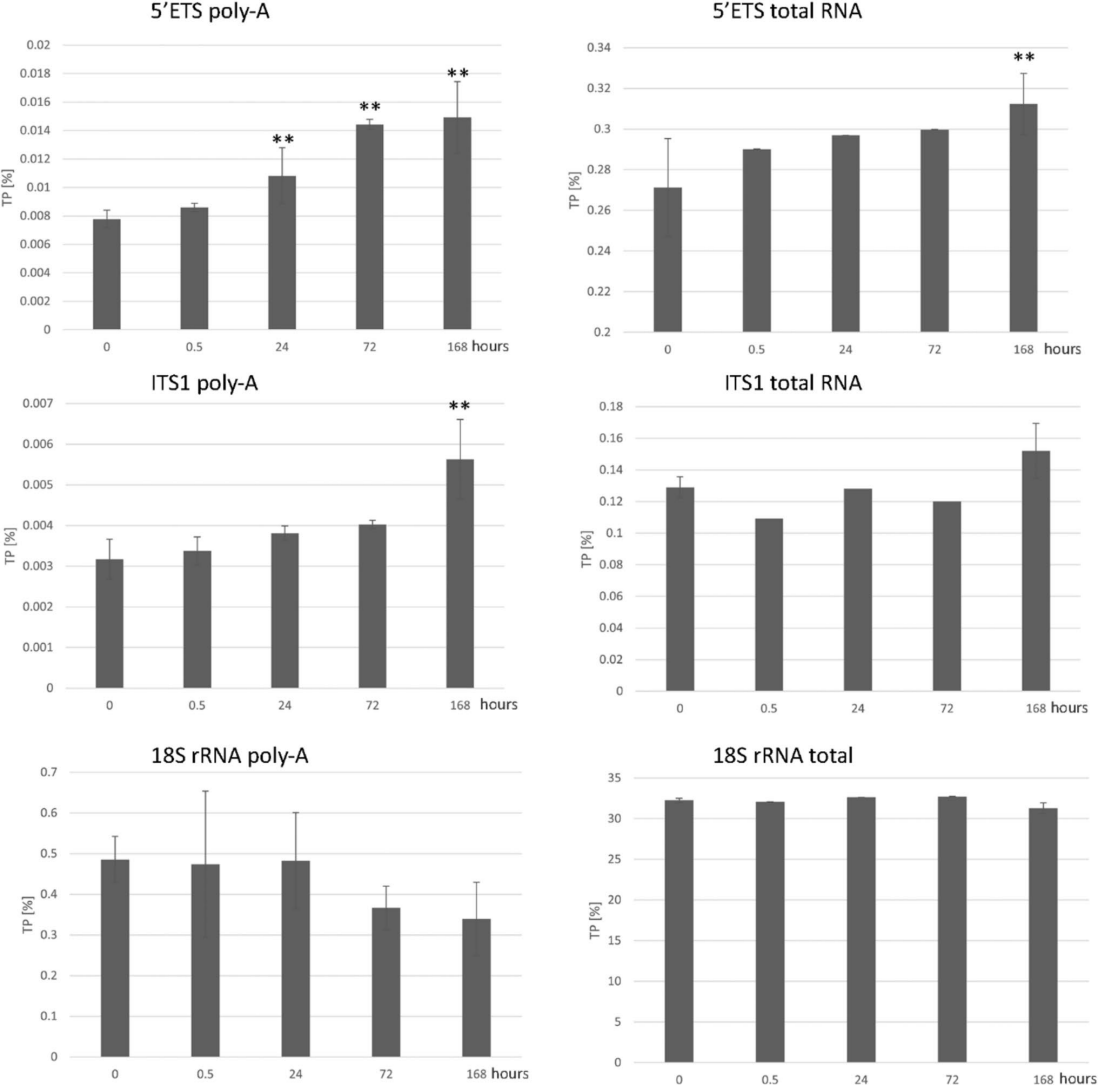
The rRNA levels in control cycling cells and following gamma irradiation. The 5’ETS, 18S rRNA and ITS1 transcripts were analyzed in poly-A (left panels) and random-primed RNA-seq (right panels) libraries. The levels are expressed as transcription proportion (TP) of mapped reads in the transcriptomes (Supplementary Table S5). The data were collected from three irradiation experiments and the TP values averaged. For control and 168-h intervals, three poly-A and three total RNA transcriptomes were evaluated. Differences between control cycling cells (interval “0”) and in cells exposed to irradiation were statistically evaluates by a two side *t* test. Significant (*p* < 0.05) differences between control and irradiated cells (different post-irradiation intervals) are indicated by “**” labels above the columns, nonsignificant differences (*p* > 0.05) have no labels

We determine the levels of coding and noncoding parts of rRNA transcripts in the course of cell transcription to senescence. To analyze the levels of different forms of rRNA transcripts we generated transcriptomic libraries derived from random (containing both polyadenylated and nonpolyadenylated RNA species) and poly-A (containing polyadenylated RNA species) primed cDNAs. Transcription proportion (TP) of individual transcripts from different rDNA subregions is shown in Fig. 6 and Supplementary Table S5. The abundance of rRNA transcripts was c. 40-fold higher in random-primed libraries as compared to the poly-A libraries.

In the poly-A fraction, the 5’ETS and 3’ETS transcripts tend to increase following irradiation reaching maximum 168 h following irradiation. The 18S rRNA levels remained stable or slightly decreased after the irradiation. In total rRNA (random library) differences were not so pronounced although increases in the levels of 5’ETS and ITS1 were also visible.

## Discussion

Nucleolus is a membrane-less organelle highly sensitive to external damage. In this study, we analyzed nucleoli integrity, expression levels of nucleolar proteins, rDNA transcription and polymorphisms following irradiation-induced replicative senescence. We found that senescence is accompanied by decreased levels of nucleolar structural proteins and increased levels of polyadenylated rRNA species. Despite altered nucleolar morphology and disbalanced of nucleolar proteins and the defects in rRNA processing key nucleolar structures remain intact in senescent cells. This may potentially contribute to radio resistance and prolonged persistence of cancer cells following radiotherapy.

### Expression of rRNA variants is not markedly influenced by the transition to senescence

We tested the hypothesis that rDNAs undergo epigenetic changes leading to altered composition of senescent ribosomes. Despite numerous high-frequency SNVs we analyzed here, the rRNA mutation profiles and frequency of variants remained similar in both cycling and senescent cells. One has to bear in mind that the mature 28S rRNA transcripts have long half life (several days), and that in nondividing senescent cells many rRNA molecules may persist from the active cycling phase. However, stable variants were detected in the 5’ETS transcripts whose half life is much shorter (minutes in cell lines (Popov et al. 2013)). This indicated the transition to senescence did not markedly influence expression of rDNA variants and that composition of senescent ribosomes is stable following the irradiation. Interestingly, most genomic variants were expressed to some extent in MCF-7 cells (Fig. 5), which resonates with cytogenetic observation that all rDNA loci were associated with the UBF transcription factor (Fig. 3). Thus, MCF-7 cells probably belong to cell lines where the most rDNA loci are associated with UBF and potentially expressed (van Sluis et al. 2020). The UBF levels have been shown to be perturbed by a number of external stresses through the mTOR signaling (Mayer and Grummt 2006). Accordingly, in our experiments, the levels of UBF were reduced in senescent cells at both protein and RNA levels (Fig. 4), indicating that the activity of Pol I might be attenuated (but not completely shut-off) following withdrawal from the cell cycle. Reduced rDNA transcription following DNA damage is also consistent with recruitment of the nSF1 silencing factor to double strand breaks in rDNA loci (Larsen et al. 2014). In short, cell senescence induced by gamma irradiation does not lead to dramatic epigenetic reprogramming of rDNA. The ribosome composition in the senescent cells is similar to cycling cells, at least regarding the 28S rRNA variants.

### Accumulation of polyadenylated rRNA species in senescent cells

One of the most profound changes associated with senescent phenotype was elevated amount of pre-rRNA transcripts, particularly in polyadenylated RNA fraction (Fig. 6). Polyadenylated forms of rRNA molecules present in minute amounts are believed to promote rRNA degradation in human cells (Slomovic et al. 2006). It may be significant that out of the different rDNA subregions, the transcripts derived from 5’ ETS and ITS1 spacers exhibit largest dynamics following the transition from cycling to senescence. Several hypotheses can be drawn:

(i) rDNAs undergo increased transcription in senescent cells. In progeria, senescent cells show higher intensity of rDNA transcription (Buchwalter and Hetzer 2017). However, we consider this possibility unlikely. First, the levels of UBF transcription factor, critical for rDNA transcription by Pol I were decreased following irradiation (Fig. 4) indicating attenuation of rDNA transcription. Second, we observed no expansion of nucleoli in senescent cells contrast to progeria. Instead, the aberrant phenotype of nucleoli resembled that of other stressors including serum starvation (Chan et al. 1985), heat shock (Liu et al. 1996), toxic chemicals (Szaflarski et al. 2022) and UV irradiation (Zatsepina et al. 1989).
(ii) Polyadenylated rRNA species may originate from RNA polymerase II (Pol II) transcription. Contrast to Pol I transcripts, most Pol II transcripts undergo polyadenylation during the export from the nucleus to cytoplasm. In Drosophila, RNA polymerase II apparently transcribed IGS leading to double strand breaks and rDNA magnification (Watase and Yamashita 2024). We cannot exclude the possibility that double strand breaks induced by gamma irradiation activated Pol II transcription of rDNA units. However, the absence of new variants in transcriptomic pools argues against this possibility. It is more likely that Pol I insufficiency causes rRNA polymerization stalling leading truncated nonproductive transcripts which end in the poly-A pathway.
(iii) Processing of rRNA transcripts might be impaired in senescence. The 5’ETS endonucleolytic cleavage in A´/01 site (Fig. 1) appear to be an early step in pre-rRNA processing particularly sensitive to stress conditions (Szaflarski et al. 2022), and indeed, most poly-A species seem to originate from that region (Fig. 6). Of note, these authors correlated early rRNA processing with nucleolar fragmentation in stressed cells induced by toxic chemicals. Alternatively, accumulation of poly-A rRNA species may reflect inhibition of poly-A export from the nucleus to cytoplasm reported in yeast (Liu et al. 1996). In contrast to yeast, we however did not observe relocation of fibrillarin from the nucleolus to cytoplasm.

## Conclusions

The data presented in this work revealed the association between nucleolar fragmentation and polyadenylated rRNA species derived from inter and intragenic spacers. A number of issues remain to be addressed since cell senescence represents a frequent outcome of organismal processes. For example, the function of intergenic spacers (IGS) which are highly variable across genera, is largely unknown. Transcripts originating from these subregions seem to modulate epigenetic state of Pol I promoters in humans (Mayer et al. 2006) and may attenuate rDNA transcription in senescence. The 5 ‘ETS is also known to bind fibrillarin which facilitates pre-rRNA processing and DFC formation (Yao et al. 2019). Fibrillarin tend to form clumped structures in senescent nucleoli (Fig. 2b) which may reflect its decreased mobility (Jo et al. 2024). It remains to be revealed what the role of polyadenylated rRNA species is in this process.

## Material and methods

### Cell culture and irradiation procedures

Human MCF-7 mammary carcinoma cell line (ATCC collection, HTB-22) was grown in Dulbecco’s modified Eagle’s medium with 10% fetal bovine serum (Gibco, Thermo Fisher Scientific), 100 U/ml penicillin, and 0.1 mg/ml streptomycin (Sigma-Aldrich). All cells were grown at 37 °C and 5% carbon dioxide (CO_2_). Irradiation was performed using a 60Co γ-ray source at Chizostat (Chirana, CR). Cells were seeded at a density of 2 × 10^5^/ml and irradiated 24 h later in culture medium at 37 °C under normal atmospheric conditions, with 8 Gy (D = 1 Gy/min). Cells were irradiated in 25 mm^2^ culture vessels for nucleic acids extractions, and on slides in four-well dishes (Nunc, #167,063, Thermo Scientific, Rochester, NY, USA) for the immunodetection. After irradiation, the cells were incubated at 37 °C and 5% CO_2_ until further treatment, with replacement of the growth medium every other day. The senescence-associated β-galactosidase (SA-*β*-gal) enzyme activity was used to monitor the onset of senescence in the course of post-irradiation interval (Senescence Detection Kit #K320-250 from Bio-Vision Incorporated (Milpitas, CA) USA) according to the manufacturer’s instructions.

### Immuno-FISH

Microscopic slides containing cells were withdrawn at different time intervals from the culture medium, washed 2 × in phosphate-buffered saline (PBS; 140 mM NaCl, 2.7 mM KCl, 1.5 mM KH2PO4, and 6.5 mM Na2HPO4; pH 7.2) at 37 °C, and fixed in 4% paraformaldehyde in PBS for 10 min at 22 °C. The cells were then rinsed briefly in PBS, washed three times for 5 min in PBS, permeabilized in 0.2% Triton X-100/PBS for 15 min at room temperature, and washed twice in PBS for 5 min. Before incubation with primary antibodies (overnight at 4 °C), the cells were blocked with 5% inactivated fetal calf serum + 2% bovine serum albumin/PBS for 30 min at room temperature. The following antibodies diluted 1:50 or 1:100 in PBS + 1% BSA were used for the immunodetection: (i) anti-UBF (mouse monoclonal, sc-13125, Santa Cruz Biotechnology, USA) and (ii) anti-fibrillarin 38F3, NB300-269, mouse monoclonal, Bio-Techne (USA). After the antibody binding, the cells in glycerol were frozen in liquid N_2_, thawed, washed 6 × in PBS, 3 × in 10 mM Tris–HCl (pH 7.5), incubated (2 h; 37 °C) with 200 μg/ml RNase A (Sigma) in 10 mM Tris–HCl (pH 7.5), washed in 10 mM Tris–HCl (pH 7.5), and then 4 × in 2 × SSC. The 220 bp-long FISH probe derived from the 3 ‘end of the human 28S rRNA gene (coordinates 12,380–12599 in the KY962518 GenBank accession) was amplified from genomic DNA using the following primers: 5 ‘-GAATTCACCCAAGTGTTGGGAT-3 ‘ (forward) and 5 ‘-AGAGGCGTTCAGTCATAATC-3 ‘ (reverse). PCR product was purified by ethanol precipitation and labelled with Cy3-dUTPs (Roche, Switzerland) in a nick translation reaction. Hybridizations were then carried out in a hybridization mixture containing formamide and 2 × SSC at 42 °C overnight, washed at a medium stringency (2 × SSC).

The immunofluorescence of the detected proteins was elicited after the incubation of microscopic samples with the Cy3-labeled second antibody to mouse IgG. Slides were mounted in Vector Shield TM mounting medium containing DAPI (4,6-diamidino-2-phenylindole) (Vector Laboratories, USA). The images were visualized using a high-resolution Leica DM RXA confocal microscope (Leica, Wetzlar, Germany) equipped with an oil immersion Plan Fluotar objective (100 ×/NA1.3) and a CSU 10a Nipkow disk (Yokogawa, Japan) for confocal imaging. A CoolSnap HQ CCD-camera (Photometrix, Tuscon, AZ, USA) and an Ar/Kr laser (Innova 70 C Spectrum, Coherent, Santa Clara, CA, USA) were used for image acquisition. In total, 100–300 cells were recorded for each set of conditions, and the experiments were repeated two or three times. The results are reported as SEM. A *t* test was used for the statistical comparison of specified samples.

### Nucleic acids extractions and high throughput sequencing

Total RNA was isolated from about 7.5 × 10^5^ MCF-7 cells using RNeasy Mini Kit (#74,104, Qiagen) according to the manufacturer’s protocol. RNA concentration was measured by NanoPhotometer N60 and the quality was checked by electrophoresis on a 0.8% agarose gel. Two kinds of cDNA libraries were prepared choosing: (i) oligo-dT primer (NEBNext^®^ Ultra II Directional RNA Library Prep Kit for Illumina, oligo-dT primer) and random primer RNA (NEBNext^®^ Ultra II Directional RNA Library Prep Kit for Illumina, random primers) for reverse transcription. The oligo-dT primer generated poly-A RNA libraries, the random primer total RNA libraries. The Illumina pair-end sequencing was carried out at Eurofins Genomics (Illumina NovaSeq 6000). After sequencing, the raw reads were filtered in Trimmomatic v.0.39 (Bolger et al. 2014). The data filtering included removing adaptor sequences, contamination and low quality reads from raw reads. The number of reads which passed thresholds for quality (Phred score > 30, > 99% reads) are given in supplementary Tab. S6.

Genomic DNA was prepared from about 1.5 × 10^6^ cells using a Macherey–Nagel™ NucleoSpin™ kit (Macherey–Nagel, Germany). DNA concentration was measured by NanoPhotometer N60. The DNA quality and integrity was checked on a 0.8% agarose gel. According to the sequencing company’s requirements, 400 ng of genomic DNA was sent to BGI (Beijing Genomics Institute, Hong Kong) for sequencing at DNBseq™ platform (DNBseq-G400). The amount of obtained cleaned (SOAPnuke software) data corresponded to about 4 × coverage.

### Bioinformatic analyses

The RNA-seq reads which mapped to repeat clusters were used to calculate the TP of repeats in the random-primed and poly-A RNA libraries. Mapping parameters were as follows: similarity fraction = 0.8, length fraction = 0.5. To calculate the transcript proportion (TP) of each gene, the number of mapped transcript reads (RNA-seq) was divided by the total number of reads and expressed as a percentage. Typically, several thousands of mapped reads were used as an input for protein coding genes; for rDNA it was hundreds of thousands. Tracks were visually inspected and checked for read coverage. Reference sequences used in mapping experiments are given in supplementary Tab. S7 and diagrammatically presented in Fig. 1. The number of cleaned reads used for SNV were called in mapped reads (ClC genomics workbench) using the following parameters: cutoff = 1 (transcriptomic reads) or 20 (genomic reads), read coverage > 200, minimum number of reads for variant call = 20. The 3’ETS transcripts could not be properly evaluated since their representation in transcriptomes was low (TP < 0.001%). Transcriptomic experiments were carried out in 2–3 biological replicates (Supplementary Tables S1 and S5).

### SDS-PAGE and Western blot

Cells were washed in PBS, scraped in the presence of Complete Mini EDTA-free protease inhibitors (Roche Diagnostics, #04693159001) and a cocktail of phosphatase inhibitors PhosSTOP (Roche Diagnostics, #04906845001) and centrifuged. Cells were washed in PBS twice and lysed in 100 µl of 1% SDS with 0,1 mM PMSF. Lysates were sonicated three times for 10 s (Bioruptor, Diagenode SA, Belgium). Protein concentration was measured both by the Bradford assay and Protein UV spectroscopy (Implen NanoPhotometer N60, Munich, Germany). Two times concentrated SDS-PAGE loading buffer was added to about fifty micrograms of total proteins of each sample. Samples were separated by 12,5% SDS/PAGE and transferred to a polyvinylidene difluoride (PVDF) membrane (#10,600,021, GE Healthcare Life Sciences, Little Chalfont, UK) using TE77XP Semi-dry blotter (Hoefer Inc., MA USA). Membranes were incubated one hour in blocking buffer TBST (20 mM Tris base, 150 mM NaCl, 0,1% Tween 20) with 1% BSA. Next, membranes were incubated over night at 4 °C in TBST buffer with following primary antibodies: antifibrillarin 38F3 (NB300-269, Novus Biologicals, Bio-Techne, USA, dilution 1:1000), anti-β-Actin (A2228, Sigma–Aldrich dilution, MA, USA, dilution 1:10,000), anti-lamin A/C (Sigma-Aldrich, #SAB4200236, dilution 1:500) and anti-UBF (mouse monoclonal, sc-13125, Santa Cruz Biotechnology, USA, dilution 1:500). Membranes were washed 6 × 5 min by TBST buffer and incubated with secondary antibody antimouse (hrp-conjugated, 1,858,413 Pierce, Thermo Fisher Scientific Inc., USA, dilution 1:3000) for one hour. Membranes were washed 6 × 5 min again and protein detection was performed using SuperSignal West Dura Extended Duration Substrate Kit (34,075, Thermo Fisher Scientific Inc., USA). The protein signals were captured using an Amersham Imager 680 (GE Health Care Life Sciences, UK).

## Supplementary Information

Below is the link to the electronic supplementary material.

Supplementary file1 (XLSX 10 KB)

Supplementary file2 (XLSX 13 KB)

Supplementary file3 (XLSX 15 KB)

Supplementary file4 (XLSX 10 KB)

Supplementary file5 (XLSX 13 KB)

Supplementary file6 (XLSX 10 KB)

Supplementary file7 (XLSX 10 KB)

## Data Availability

Sequences generated within this study are available in GenBank SRA archive under the Bioproject PRJNA1221647.
